# Obinutuzumab-induced severe acute thrombocytopenia: a case report and literature review

**DOI:** 10.3389/fimmu.2025.1609862

**Published:** 2025-08-21

**Authors:** Kun Kou, Qiaolin Zhou, Lijun Du, Fang Xu

**Affiliations:** Department of Haematology, Mianyang Central Hospital, School of Medicine, University of Electronic Science and Technology of China, Mianyang, China

**Keywords:** follicular lymphoma, obinutuzumab, severe, acute, thrombocytopenia

## Abstract

Obinutuzumab is a humanized type II anti-CD20 monoclonal antibody that is widely used in B-cell lymphomas including follicular lymphoma (FL) and chronic lymphocytic leukemia (CLL). Multiple clinical studies have shown that compared with rituximab combined with chemotherapy, obinutuzumab combined with chemotherapy can significantly improve the progression-free survival (PFS) of patients, effectively reduce the risk of disease progression, and improve patient prognosis. The main adverse effects of obinutuzumab include infusion reactions, myelosuppression, infection, cardiotoxicity, tumor lysis syndrome (TLS), etc., and in rare cases it may induce thrombocytopenia. However, so far there are few reports on “obinutuzumab-induced acute thrombocytopenia” (OIAT), especially severe cases. Here, we report a case of acute severe OIAT and review the literature to explore the management of this rare but life-threatening complication. The case is a 28-year-old young man who was diagnosed with stage IV follicular lymphoma and achieved remission after 8 cycles of R-CHOP chemotherapy. Later, he developed severe acute thrombocytopenia during maintenance treatment with obinutuzumab monotherapy, the patient’s platelet count dropped from 191×10^9/L to 2×10^9/L on the 3rd day after the initial application, and severe thrombocytopenia occurred after multiple subsequent applications of obinutuzumab. OIAT is a rare but life-threatening complication. We should be aware of this adverse event and raise awareness about it.

## Introduction

Obinutuzumab is a humanized type II anti-CD20 monoclonal antibody commonly used to treat follicular lymphoma (FL) and chronic lymphocytic leukemia (CLL) (1–4). Compared with type I anti-CD20 monoclonal antibodies such as rituximab, the glycosylation modification of the Fc segment of obinutuzumab can enhance its affinity with immune effector cells, resulting in stronger antibody-dependent cellular cytotoxicity (ADCC), better direct B cell killing effect (DCD), lower complement-dependent cytotoxicity (CDC) (5, 6). These pharmacological properties lower immunogenicity risk, effectively alleviate the problem of rituximab resistance, and further improve efficacy. As a macromolecular compound, the main adverse reactions of obinutuzumab include infusion reactions, myelosuppression, infection, cardiotoxicity, tumor lysis syndrome (TLS), etc (7–9). A few early clinical studies reported obinutuzumab-related platelet cytopenia (10–12), however, obinutuzumab-induced acute thrombocytopenia (OIAT), especially severe cases, are rarely reported. Management of OIAT is challenging due to the lack of specific guidelines. Here, we report a case of severe acute thrombocytopenia in a patient following an infusion of obinutuzumab. We also review the literature on this rare but life-threatening side effect. Using PubMed, we searched for articles published between 2014 and 2024 that contained the words “obinutuzumab,” “thrombocytopenia,” “severe,” and “acute.”

## Case report

This patient was a 28-year-old man who inadvertently discovered enlarged lymph nodes in the right neck. The condition gradually progressed and affected the bilateral neck, armpits, groin and other areas. During the disease, the number and volume of lymph nodes significantly increased. In 2021, he was diagnosed with pathological grade I follicular lymphoma with involvement of pleura and bone marrow, stratified with stage IV, FLIPI-2 score 3 and high risk. This 28-year-old male patient had no prior diagnosis of systemic diseases. Specifically, there was no evidence of autoimmune disorders or hematologic diseases in his medical records. Additionally, he reported no history of chronic medication use or surgical procedures. At diagnosis, baseline complete blood count showed white blood cells 5.53×10^9^/L, hemoglobin 119 g/L, and platelets 121×10^9^/L. The patient had no history of underlying diseases such as thrombocytopenia, hemorrhagic disorders, or autoimmune diseases. Then the patient underwent 8 cycles of R-CHOP regimen (rituximab 600 mg D0, cyclophosphamide 1.2 g D1, doxorubicin liposomal 60 mg D1, vincristine 2 mg D1, prednisone acetate 70 mg D1-5), throughout the chemotherapy course, platelet counts remained stable within the normal range (100-300×10^9^/L). Post-treatment PET/CT evaluation demonstrated complete metabolic response. Based on the results of the GALLIUM trial, which demonstrated superior progression-free survival (PFS) with obinutuzumab maintenance therapy compared to rituximab in follicular lymphoma (FL) (8, 13), particularly in high-risk subgroups,we selected obinutuzumab (1000 mg administered every 8 weeks) as the maintenance regimen. Although hematologic toxicities (e.g., thrombocytopenia) were observed, long-term follow-up data confirmed that the survival benefits of obinutuzumab outweighed these manageable risks. The result of routine blood test the day before the initial treatment showed that the platelet count was normal at 191×10^9/L. On the third day after obinutuzumab treatment, the platelet count decreased to 2×10^9/L, while the white blood cell and haemoglobin counts were normal. The platelet count recovered to be normal at 176×10^9/L on the day before the second maintenance treatment of obinutuzumab. The platelet count dropped to 4×10^9/L on the first day after the treatment again. The platelet counts before the third maintenance treatment with obinutuzumab was 152×10^9/L, and the platelet count dropped to 1×10^9/L on the third day after the end of treatment. During the period of thrombocytopenia, the patient manifested with ecchymoses and petechiae on the skin and mucous membranes of the limbs, without active bleeding. After platelets transfusion, cortisol hormones, interleukin-11 and other treatments, the patient’s thrombocytopenia lasted about 2 months and recovered to normal levels before subsequent maintenance treatment. Due to the repeated episodes of acute severe thrombocytopenia after obinutuzumab treatment, the maintenance treatment regimen was adjusted as rituximab in the fifth cycle and no thrombocytopenia recurred.

## Discussion

Obinutuzumab, a novel CD20 monoclonal antibody, has been verified with significant efficacy by several key clinical studies, especially in prolonging progression-free survival (PFS) as well as reducing the risk of recurrence in the treatment of follicular lymphoma (FL) (8).However, the application of any innovative drug may be accompanied by potential side effects, among which thrombocytopenia was reported to be one of the rare adverse reactions during the treatment of obinutuzumab (10, 11).The mechanism of obinutuzumab caused thrombocytopenia is currently unclear. Herein, case reports of acute severe thrombocytopenia induced by obinutuzumab are relatively rare, only five definite cases of OIAT reported in the literature (**Table 1**), and a variety of potential mechanisms were supposed to be involved.

**Table 1:** Summary of all published cases reporting OIAT.

Long-term Complications	None	None	None	None	None	None
IVIG Dose(if used)	Not used	10g/day × 3days	Not used	Not used	Not used	Not used
Recovery Time(Days)/Level	7 days / Partial recovery(to grade 1)	6 days / Complete recovery (normal)	10 days / Complete recovery (normal)	9 days / Partial recovery(to grade 1)	20 days / Partial recovery(to grade 2)	9 days / Partial recovery(to grade 3)
Management	Platelet transfusions , intravenousimmunoglobulins	Platelet transfusions , IVIG	Platelet transfusion	Platelet transfusions, TPO-RA, steroids	Platelet transfusions, TPO-RA, steroids	Platelet transfusion,Interleukin-11,steroids
Platelet nadir afterTreatment(×10^9/L)	40	4	21	3	3	2
Platelet count beforeTreatment(×10^9/L)	150	245	160	376	76	191
Disease	CLL	FL	FL	FL	MCL	FL
Age/gender	68y/ Female	56y/Female	64y/Female	74y/Female	44y/Female	28y/Male
Reference	Walter et al., 2016 (19)	Haage et al., 2022 (11)	Sakai et al.2020 (20)	Mechelfekh et al.2023 (10)	Mechelfekh et al.2023 (10)	Current report

## Mechanism 1:complement activation and cytokine cascade

The glycosylated Fc segment of obinutuzumab is designed to enhance binding to FcγRIIIa on immune effector cells (e.g., NK cells, macrophages), thereby improving antitumor efficacy (5, 8, 14). However, this potent activation may unexpectedly trigger the complement cascade, initiated by C1q binding, leading to massive release of inflammatory cytokines such as IL-6 and TNF-α. As proposed by Aster and Bougie (15), complement fragments (C3b, C5a) can opsonize platelets, promoting their clearance through complement-dependent cytotoxicity (CDC) and macrophage phagocytosis. This complement-mediated acute destruction pattern is evidenced by both the rapid onset in our case (platelets dropping to 2×10^9/L within 72 hours after the first dose) and the case reported by Haage et al. (platelets declining to 4×10^9/L within 24 hours post-administration) (11). Notably, the marked efficacy of IVIG in Haage’s case further supports this mechanism (11), as IVIG exerts its therapeutic effect by neutralizing complement fragments and blocking Fcγ receptors.

## Mechanism 2:FcγR-mediated platelet clearance

The type II structure of obinutuzumab enables stronger antibody-dependent cellular cytotoxicity (ADCC) and weaker complement-dependent cytotoxicity (CDC) compared to type I anti-CD20 antibodies like rituximab (5, 6). This distinct property may facilitate platelet-specific ADCC in susceptible individuals: immune effector cells recognize obinutuzumab-bound platelets via FcγRIIIa, leading to platelet destruction (16). Additionally, obinutuzumab binding may directly engage FcγRIIa receptors on platelet surfaces, triggering intracellular caspase-3-dependent apoptosis and accelerating programmed cell death (5, 17, 18). Sakai et al. experimentally confirmed this FcγRIIa-mediated pro-apoptotic effect. During the first episode of OIAT, the patient we reported received hormonal therapy for one consecutive week, interleukin-11 therapy for 3 days and one therapeutic dose of platelet transfusion, until platelet counts increased from 2×10^9/L to 48×10^9/L. However, during the second episode of OIAT, the patient received 10 days of interleukin-11 treatment without hormone therapy, the platelet grew significantly slowly. After 11 days of treatment, the platelet counts only increased from 4×10^9/L to 18×10^9/L. After the third application of obinutuzumab, the platelet count dropped sharply to 1×10^9/L again. In this episode, the patient received 8 days of interleukin-11 combined with 6 days of prednisone acetate. After 8 days of treatment, the platelet count significantly increased to 74×10^9/L. In this patient, the use of steroids enhanced the effects in raising platelet counts. The efficacy of glucocorticoids in our case further supports this mechanism, as steroids inhibit macrophage activity and downregulate FcγR expression to block phagocytosis.

## Mechanism 3:drug-dependent antibody mechanism

Drug-dependent antibodies (DDAs) represent a core mechanism in classic drug-induced thrombocytopenia, such as heparin-induced thrombocytopenia (15). Unlike conventional autoantibodies, DDAs are neoantibodies generated after drug exposure—they specifically recognize cryptic epitopes on platelet membrane glycoproteins (e.g., GPIIb/IIIa) only when the drug (e.g., obinutuzumab) is present, thereby triggering macrophage-mediated phagocytosis (15). Although obinutuzumab is a humanized antibody, its macromolecular protein structure still carries a potential risk of inducing DDAs (5).The most distinctive feature in our case was the”re-exposure consistency” of thrombocytopenia—severe platelet depletion recurred rapidly (within days) following each of the three obinutuzumab administrations. This highly reproducible pattern of rapid platelet destruction upon repeated drug exposure is a characteristic feature of DDA-mediated thrombocytopenia, as seen in heparin-induced thrombocytopenia. It strongly suggests the involvement of an adaptive immune response involving immune memory, where prior exposure has primed the immune system to mount a swift and consistent reaction upon re-encountering the drug.Although direct evidence of DDAs in obinutuzumab-induced acute thrombocytopenia (OIAT) remains unavailable, this phenomenon strongly warrants future DDA screening in similar cases to validate the mechanistic link between drug re-exposure and immune memory amplification.

Notably, complement activation, FcγR-mediated platelet destruction, and potential drug-dependent antibody (DDA) involvement are not mutually exclusive mechanisms. These pathways may act synergistically within individual patients, collectively driving rapid platelet depletion.

Based on the established mechanisms and existing research, future diagnostic strategies may investigate disease etiology through three key approaches: dynamic monitoring of complement activation markers (including serum C3a, C5a, and sC5b-9), measurement of cytokine levels (such as IL-6 and TNF-α), and screening for drug-dependent antibodies (DDAs) using the MAIPA assay to detect obinutuzumab-dependent antiplatelet antibodies targeting epitopes like GPIIb/IIIa or GPIb/IX (5, 15).

Currently, there is a lack of consensus on treatment and management of OIAT. Therefore, we reviewed the 6 cases of OIAT reported up to now. Six cases are listed in **Table 1**, of which 5 were female and 1 was male. Systematic analysis of six OIAT cases revealed that all patients developed severe thrombocytopenia (nadir: 2–40×10^9^/L) within 72 hours post-obinutuzumab infusion, necessitating intensive platelet monitoring during the first treatment week. Treatment responses exhibited significant divergence: immunomodulatory therapies (IVIG/corticosteroids) accelerated recovery efficiently. Haage et al.’s case achieved complete platelet recovery within 6 days using IVIG (11), while our case demonstrated faster platelet elevation with corticosteroid-IL-11 combination versus IL-11 monotherapy. In contrast, thrombopoiesis-stimulating agents (TPO-RAs, IL-11) showed limited efficacy, supporting an immune-mediated destruction mechanism rather than production deficiency. Notably, 83.3% (5/6) of cases involved high-risk B-cell malignancies,including follicular lymphoma (FL, n=4), chronic lymphocytic leukemia (CLL, n=1), and mantle cell lymphoma (MCL, n=1), suggesting potential risk amplification by high tumor burden. OIAT occurred in both chemotherapy-combination groups (e.g., bendamustine in Haage’s case (11)) and monotherapy groups, excluding pure chemotherapy-induced myelosuppression. Critically, obinutuzumab re-exposure yielded a 50% (2/4) recurrence rate, whereas all patients switching to rituximab (2/2) maintained remission. Based on these clinical characteristics and differences in treatment responses, combined with existing treatment experience, we have developed a reference treatment decision-making protocol for OIAT (**Table 2**). The initiation criteria and drug dosage recommendations of this protocol primarily derive from the clinical data of these 6 limited cases and the recommendations outlined in guidelines for immune thrombocytopenia (ITP). It is important to emphasize that, given that the pathogenesis of OIAT has not yet been fully elucidated, treatment regimens need to be individually adjusted according to the specific conditions of each patient in clinical practice. For patients with concurrent infections, hepatic or renal insufficiency, or a high risk of thrombosis, in particular, careful evaluation of drug selection and dosage adjustments is required, along with close monitoring of treatment responses and adverse reactions.

**Table 2:** Clinical decision matrix for thrombocytopenia management.

Intervention measures	Initiation criteria	Recommended regimens	Sources of evidence
Glucocorticoids	Platelet count <30×10^9^/L or presence of active bleeding	Methylprednisolone 0.5-2 mg/kg/d; Dexamethasone 40 mg/day × 4 days; Prednisone 1 mg/kg/d (max 80 mg/d)	Current report
IVIG	Glucocorticoid intolerance/contraindication; urgent intervention required; or platelet count <10×10^9^/L	0.4 g/kg/d × 5 days; 10 g/day × 3 days	Haage et al., 2022 (11)
TPO-RA	Platelet count <50×10^9^/L	Romiplostim 10 μg/kg/week; Eltrombopag 25-75 mg/day	Mechelfekh et al.2023 (10)
IL-11	Not recommended for monotherapy	–	Current report

Based on the treatment decision-making framework (**Table 2**), this study further proposes a stratified management strategy to optimize the timing of clinical interventions and drug selection. Permanent discontinuation of obinutuzumab is mandatory upon detection of platelet counts below 50×10^9^/L or active bleeding. For patients in the acute phase with platelet counts below 30×10^9^/L, initiating intravenous immunoglobulin (IVIG) within 24 hours is recommended. IVIG is particularly critical for patients presenting with platelets below 10×10^9^/L or concurrent bleeding, as a complete platelet recovery within 6 days has been reported (11). When IVIG is unavailable, corticosteroid therapy represents an alternative option; platelet transfusion is also necessary for patients experiencing active bleeding.Thrombopoietin receptor agonists (TPO-RAs) or interleukin-11 (IL-11) are strictly reserved as adjunctive therapy during the subacute phase (>72 hours) for patients exhibiting persistent platelet counts below 30×10^9^/L. This restriction is due to the delayed onset of action of these agents, with only partial platelet recovery observed even after 9-20 days (10).

Risk stratification is essential when considering anti-CD20 monoclonal antibody (mAb) rechallenge. Rechallenge is contraindicated in patients with platelet counts <10×10^9^/L (50% recurrence rate), previous rechallenge failure, or hemorrhagic complications. For these individuals, permanent substitution with an alternative CD20 mAb (e.g., rituximab) is mandatory. Prognostic analysis confirms that platelet recovery occurs in all patients after obinutuzumab discontinuation. Furthermore, no disease recurrence was observed in patients transitioning to rituximab, supporting the necessity and safety of this therapeutic adjustment. In patients with platelet counts ≥10×10^9^/L and no active bleeding, cautious rechallenge may be considered under prophylactic intravenous immunoglobulin (IVIG) coverage with daily monitoring. However, the high recurrence risk (50%, evidenced by failure in 2/4 rechallenge cases from Mechelfekh et al. and the current study) must be explicitly communicated prior to rechallenge.

To advance research on obinutuzumab-induced thrombocytopenia (OIAT), future studies should prioritize establishing an international multicenter registry system through pharmacovigilance networks (e.g., FDA FAERS or EU EudraVigilance) to standardize global case data collection. This system should include core datasets on demographic characteristics (age, lymphoma subtypes), treatment parameters (dose, infusion rate), platelet dynamics (nadir, recovery time), and therapeutic responses (efficacy of IVIG/corticosteroids), enabling accurate incidence estimation and high-risk population identification. Concurrently, prospective cohort studies are needed to screen treatment-naïve patients for complement activation markers (C3a, C5a, sC5b-9) and FcγRIIIa polymorphisms (V158F) before infusion, while post-infusion monitoring must incorporate serial platelet counts and drug-dependent antibody (DDA) titers measured via modified MAIPA assay. These data will support early-warning models, such as predicting severe risk when platelet counts drop >50% within 24 hours. Building on these foundations, mechanism-driven stratified trials should be implemented: for complement-activated subtypes (C3b deposition >2× baseline), validate anti-C5 monoclonal antibodies (e.g., eculizumab) combined with IVIG; for DDA-positive subtypes, employ IVIG plus corticosteroids; and for complement-dominant subtypes, utilize anti-C5 monotherapy. Real-world data integration is essential to develop EHR-embedded alert tools using NLP to identify acute cases (keywords: obinutuzumab + thrombocytopenia + acute onset), facilitating re-exposure risk stratification where low-risk patients receive premedication with slowed infusions while high-risk patients permanently switch to rituximab. Finally, patient empowerment via mobile health tools (e.g., OIAT-specific apps) should guide bleeding documentation and portable platelet self-testing during the critical 24-72-hour post-infusion window, completing a closed-loop framework of “mechanistic dissection - risk prediction - precision intervention - patient engagement”. This case report of thrombocytopenia in a follicular lymphoma patient after taking obinutuzumab reminds us once again that we need to pay attention to potential side effects when using new targeted drugs.

OIAT is a rare and potentially life-threatening adverse reaction, the pathophysiological mechanism of which is not fully understood. Close monitoring of bleeding symptoms and platelet counts early after the first infusion of Obinutuzumab are recommended. Currently, there is a lack of consensus guidelines for its treatment. Due to the recurrence risks of OIAT and no systemic cross-toxicity, rituximab can be considered in the future once patients develop severe thrombocytopenia.

## Data Availability

The raw data supporting the conclusions of this article will be made available by the authors, without undue reservation.
